# Oleocanthal, a Phenolic Derived from Virgin Olive Oil: A Review of the Beneficial Effects on Inflammatory Disease

**DOI:** 10.3390/ijms150712323

**Published:** 2014-07-11

**Authors:** Lisa Parkinson, Russell Keast

**Affiliations:** School of Exercise and Nutrition Sciences, Centre of Physical Activity and Nutrition Research (CPAN), Deakin University, 221 Burwood Highway, Burwood, VIC 3125, Australia; E-Mail: ljlu@deakin.edu.au

**Keywords:** virgin olive oil, ibuprofen, sensory, inflammation, health

## Abstract

Virgin olive oil (VOO) is credited as being one of many healthful components of the Mediterranean diet. Mediterranean populations experience reduced incidence of chronic inflammatory disease states and VOO is readily consumed as part of an everyday dietary pattern. A phenolic compound contained in VOO, named oleocanthal, shares unique perceptual and anti-inflammatory characteristics with Ibuprofen. Over recent years oleocanthal has become a compound of interest in the search for naturally occurring compounds with pharmacological qualities. Subsequent to its discovery and identification, oleocanthal has been reported to exhibit various modes of action in reducing inflammatory related disease, including joint-degenerative disease, neuro-degenerative disease and specific cancers. Therefore, it is postulated that long term consumption of VOO containing oleocanthal may contribute to the health benefits associated with the Mediterranean dietary pattern. The following paper summarizes the current literature on oleocanthal, in terms of its sensory and pharmacological properties, and also discusses the beneficial, health promoting activities of oleocanthal, in the context of the molecular mechanisms within various models of disease.

## 1. Introduction

The health promoting attributes associated with following a traditional Mediterranean diet have been recognised for decades, with the first suggestion of healthful effects accompanying the Seven Countries Study [1]. The risk of chronic inflammatory disease in Mediterranean populations are the lowest in the world, and life expectancy amongst the highest [2] which has earned the populations residing along the Mediterranean sea considerable attention from nutrition researchers worldwide. Since the inaugural Seven Countries Study numerous other studies have supported the view that this pattern of eating is associated with a reduced incidence of inflammatory disease states [3,4,5,6,7,8,9,10,11].

The principle source of dietary fat in the Mediterranean diet is virgin olive oil (VOO) [12] and this has in part been recognised as a contributing factor towards the favourable health profile that the Mediterranean population possess [13]. Throughout history VOO has been recognized as valuable pharmacological agent in the hands of ancient Greek doctors [14]. Hippocrates mentions approximately 60 health conditions where VOO use can be beneficial, for example many skin conditions, wounds and burns, amongst others. Typically traditional Mediterranean populations consume 25–30 mL of oil as part of their diet, generally in salad dressings as well as cooked foods [15].

Importantly VOO contains approximately 36 phenolic compounds, and it is this minor phenolic fraction of VOO that is partially responsible for the health benefits that accompany intake [16,17,18]. An expanding volume of studies (including human, *in vivo* and *in vitro*) have reported that VOO phenolics have beneficial effects on inflammation, antioxidant status, antimicrobial activity, as well as other biological markers of non- communicable disease. (for review see [18]).

A phenolic compound in VOO that stands alone in terms of sensory and anti-inflammatory attributes is decarboxy methyl ligstroside aglycone (also known as oleocanthal) [19]. Oleocanthal is homologous with the non-steroidal anti-inflammatory drug (NSAID) ibuprofen [19] for both perceptual and anti-inflammatory properties. Both compounds produce a localised irritation in the oropharangeal region and further research instigated, because of the perceptual similarities, found that oleocanthal shares a similar anti-inflammatory action with Ibuprofen [19] therefore oleocanthal is now acknowledged as a naturally occurring NSAID [20,21,22,23,24].

Importantly it has been proposed that the long term daily ingestion of virgin olive oil (containing oleocanthal), which is a common attribute of the traditional Mediterranean diet, may be partially responsible for the favorable health profile of Mediterranean populations [19]. Chronic low doses of ibuprofen and other COX inhibitors such as aspirin have been shown to have anti-carcinogenic and anti-thrombotic effects [25,26,27]. Therefore, it is plausible that low, chronic doses of a naturally occurring NSAID such as oleocanthal may attenuate inflammation over time, and may then contribute to significant reductions in the development of chronic inflammatory disease.

## 2. Oleocanthal Identification

Oleocanthal was first documented in literature as a phenolic compound contained in VOO in the early 90’s [28]. It was a decade later that the compound, then known as decarboxy methyl ligstroside aglycone, was suggested to be the sole phenolic responsible for the distinct throat irritation and pungency elicited by some VOOs [29]. Beauchamp and colleagues, substantiated this finding over a decade later reporting that decarboxy methyl ligstroside aglycone was the sole irritant phenolic responsible for the peppery stinging sensation experienced with VOO ingestion and aptly named the compound oleocanthal (*oleo* for olive, *canth* for sting, and *al* for aldehyde) [19].

The confirmatory finding that oleocanthal is the sole irritating compound in VOO was achieved by quantifying oleocanthal from various VOO’s and measuring the throat irritation accompanying ingestion. However, to exclude the possibility that other compounds in VOO may also contribute to the unique perceptual characteristic, oleocanthal was synthesised and dissolved in corn oil. The measure of the throat irritation from the addition of oleocanthal to non-irritating corn oil was found to be dose dependent on oleocanthal and mimicked the irritation of VOO [19] confirming that indeed oleocanthal is the sole compound responsible for throat irritating sensation.

A point of interest is that the spatial irritation produced by oleocanthal is specific to the oropharyngeal region. Generally irritant or pungent compounds are perceived in all regions in the oral cavity, rather than isolated to a spatially distinct area. This implies that a sensory receptor specific to oleocanthal exists in the oropharyngeal region [30]. Peyrot des Gachons and colleagues [31] have identified the Transient receptor potential cation channel, subfamily A, member 1 (TRPA1) as the receptor linked to oleocanthal. This large inter-individual variation in sensitivity to oleocanthal may then be due to variation in expression of TRPA1 receptors in the oropharyngeal region.

## 3. Sensory and Anti-Inflammatory Profile of Oleocanthal

A key characteristic of VOO quality is pungency and irritation. These attributes are recognised as the positive reinforcement of VOO quality by those who frequently consume the oil, such as Mediterranean populations. So much so, that prized VOO’s are rated as one cough or two cough oils, with the latter classed as superior [31]. There is reported variation in the concentration of oleocanthal contained in VOO. A recent study has reported that the concentration of oleocanthal contained in VOO ranges from 284 to 711 mg/kg in a variety of Greek oils [32] and similar variance was also reported in an earlier study [33].

Although the concentration of oleocanthal differs amongst oils and may influence irritant properties of the oil [34], there is another key factor. The degree in which an individual experiences bitterness or irritation of a compound is determined by an individual’s sensitivity to the compound, and large inter-individual variation in the perceived intensity of irritation of oleocanthal has been reported. Cicerale and colleagues [30] reported that intensity ratings of irritation to a oleocanthal concentration of 54 mg/kg, contained in the VOO matrix, ranged from a slight irritation in the throat, to an irritation that was of intensity sufficient to produce a cough in those highly sensitive.

**Figure 1:**
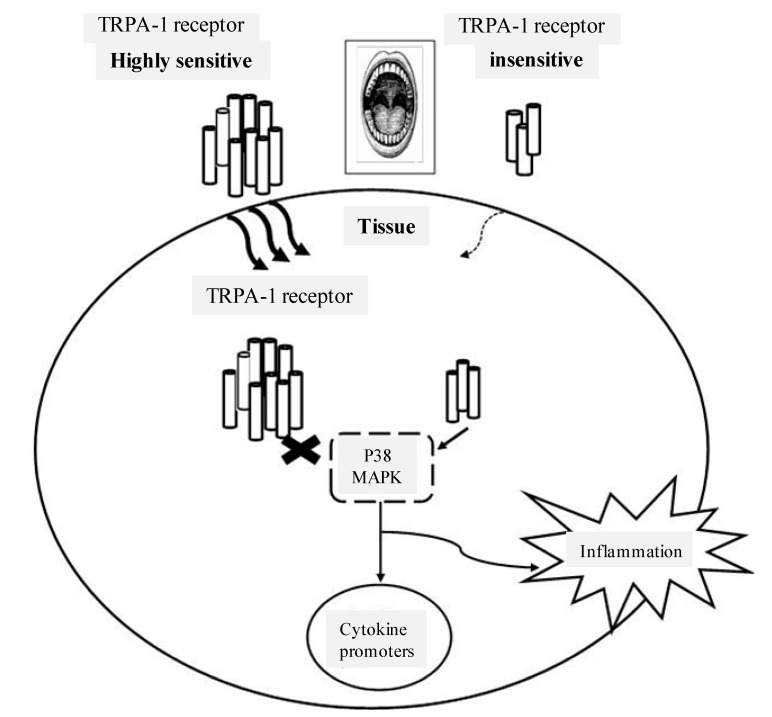
It is possible that receptor levels of TRPA-1 in muscle reflect levels in the oropharyngeal region. Therefore low or high levels of TRPA-1 in the oropharyngeal region may reflect the inflammatory responses in muscle through activation of P38 MAPK.

Fischer and colleagues hypothesised that similar perceptual properties may be reflective of similar pharmacological properties as early as 1965, suggesting that the more pronounced the perceptual properties, the more potent the pharmacological properties [35]. For example, the more bitter tasting a compound is rated, the more potent the anti-inflammatory, anti-oxidant, or anti-microbial actions of that compound may be. This may have important implications for investigations into suitable naturally occurring NSAIDs. If levels of TRPA-1 are expressed in a coordinated manner throughout the body, and greater expression results in higher oropharyngeal irritation, then perhaps muscle and other tissue would also have high expression levels of TRPA-1 receptor. To elaborate, oleocanthal sensitivity is linked to variation in oropharyngeal TRPA1 levels, and TRPA-1 is activated in response to inflammatory cytokines. Therefore differences in oral sensitivity may indicate how oleocanthal influences inflammatory pathways in muscle (Figure 1). Future investigations may determine if oleocanthal differentially attenuates inflammation based on individual differences in perceptual sensitivity to the oropharyngeal irritation elicited by this phenolic compound.

After the perceptual similarity between oleocanthal and ibuprofen were confirmed, Beauchamp and colleagues [19] explored the possibility that the pathway of anti-inflammatory activity was also analogous between compounds. The results of the study clearly show that oleocanthal inhibits cyclooxygenase 1 and 2 (COX 1 & 2) enzymes in a dose-dependent manner, and does in fact mimic the anti-inflammatory action of the synthetic NSAID ibuprofen. The important and novel findings of Beauchamp and colleagues [19] demonstrate that oleocanthal not only mimics the mode of ibuprofen inflammatory activity, but inhibits COX 1 and COX 2 enzymes significantly more at equimolar concentrations. For example, oleocanthal (25 µM) inhibits 41%–57% of COX activity in comparison to ibuprofen (25 µM) which inhibits 13%–18% of COX activity. This adds further weight to oleocanthal as a potential factor in the health benefits associated with a traditional Mediterranean Diet. Assuming approximately 70% absorption, then 50 mL/day corresponds to approximately 10% the current Ibuprofen pain relieving dose. This estimate differs according to oleocanthal concentrations in VOO.

## 4. Oleocanthal and Cancer

Numerous *in vitro* studies have reported that the phenolic compounds in VOO can inhibit the initiation and metastasis of several types of cancer. This in turn supports the abundance of associative evidence highlighting the lower incidence of many types of cancer, including breast, prostate, lung and gastrointestinal cancer that are observed in Mediterranean populations when compared to Western populations [36,37,38,39,40]. The inflammatory enzymes attenuated by oleocanthal, COX 1 and COX 2, are responsible for the conversion of arachidonic acid to prostaglandins and thromboxane, which are produced in response to inflammatory or toxic stimuli [41]. One cyclooxygenase enzyme, COX 2 is implicated in the pathogenesis of several cancers, in both human and animal studies [42,43,44,45,46]. Because oleocanthal is a naturally occurring COX inhibitor, it is becoming a compound of interest in cancer research.

Recent evidence highlights oleocanthal as therapeutic medium by which monocyte chemoattractant protein 1 (MIP-1α) is attenuated in a human multiple myeloma cell line (ARH-77). MIP-1α is reported to be a critical instigator of malignant lesions in bone marrow milieu [47]. Additionally, Khanal and colleagues [48] have recently demonstrated that oleocanthal exerts an anti-proliferative effect, and therefore prevents tumour induced cell transformation in mouse epidermal JB6 Cl41cells. Oleocanthal accomplishes this via the inhibition of extracellular signal-regulated kinases 1/2 and p90RSK phosphorylation. Furthermore, oleocanthal encourages cell apoptosis by activating caspase-3 and poly-adenosine diphosphate-ribose polymerase, phosphorylates p53 (Ser15), and also induces the fragmentation of DNA in HT-29 cells derived from human colon adenocarcinoma [48]. In addition to this it would seem from recent observations that oleocanthal potentially disrupts the pathogenesis of c-Met kinase related malignancies. Elnagar and colleagues [22] have reported that oleocanthal has significant anti-proliferative effects that have been demonstrated in both human breast and prostate cancer lines. Additionally data from this study showed that oleocanthal exhibits anti-migratory and anti-invasive actions in PC-3 and MDA-MB-231 cells possibly as a result of its ability to inhibit c-Met phosphorylation [22].

Further to this, recent research has unveiled an unusual effect of oleocanthal on heat shock protein 90 (Hsp90) [49]. Hsp90 is a chaperone protein that stabilizes a number of proteins that are required for tumor growth. Therefore Hsp90 inhibitors are investigated as anti-cancer drugs. Margarussi and colleagues report that oleocanthal significantly reduce two Hsp90 proteins, Akt and Cdk4 without actually influencing Hsp90 regulation in 2013. Furthermore oleocanthal had a pro-apoptotic effect on cancer cells but only a slight effect on the viability of peripheral blood mononuclear cells, which is a common attribute of Hsp90 inhibitors. It would therefore seem that oleocanthal may be a compound of interest in regards to endeavours to identify a new class of Hsp90 inhibitors [49], and is a compound of importance in future cancer research.

## 5. Oleocanthal and Joint-Degenerative Disease

Inflammation plays a significant role in the pathogenesis of joint degenerative disease and given the prevalence of this disease it is important to identify suitable pharmacological interventions. Oleocanthal has recently been highlighted as a therapeutic compound that may be of interest in the quest to find suitable natural NSAIDs for the treatment of joint degenerative disease. Pro-inflammatory cytokines stimulate nitric oxide (NO) production [50], up-regulate the synthesis of cartilage degrading enzymes, as well as increase prostaglandin PGE_2_ production, all factors that are implicated in the development of joint-degenerative disease. In osteoarthritis (OA) pathogenesis, diseased cartilage synthesizes NO spontaneously from diseased chrondocytes [51]. NO plays a pivotal role in joint-degenerative disease and the stable end product of NO, nitrite (NO_2_), is significantly expressed in arthritic synovial fluid [20]. NO is biosynthesized by nitric oxide synthase (NOS). Another form of NOS is inducible NOS (iNOS) which is primarily responsible for the inflammatory actions of NOS [52]. Iacano and colleagues reported that oleocanthal and synthesized derivatives, attenuate production of iNOS protein expression in an LPS challenged murine chondrocytes, in a dose dependent manner.

Also, as oleocanthal inhibits COX enzymes, and prostaglandins are downstream of COX then it is possible that oleocanthal may also exert pharmacological actions in the treatment of both rheumatoid arthritis and osteoarthritis through COX inhibition. COX enzymes are a catalyst for the formation of prostaglandins and prostaglandins are highly expressed in the arthritic spine in an animal model [53]. Therefore, oleocanthal may attenuate arthritic pain through inhibition of prostaglandins, specifically the synthesis of PGE_2_ that accompanies COX inhibition.

Inflammatory mediators such as Interleukin 6 (IL-6) and (MIP-1α) have been associated with osteoarthritis (OA) and rheumatoid arthritis (RA) [54,55] and accordingly NSAIDs are often prescribed, however with considerable adverse effects, such as damage to gastrointestinal mucosa [56]. Scotece and colleagues (2012) investigated the effects of oleocanthal exert on potential targets of joint inflammation reporting that oleocanthal inhibits NO production in J774 macrophages and inhibits both IL-6 and MIP-1α in both ATDC5 chrondocytes and J774 macrophages [57]. These inflammatory cytokines are both implicated in the inflammatory process and cartilage destruction of inflammatory arthropathies. Further to this, oleocanthal decreased expression of other pro-inflammatory markers Interleukin 1 (IL-1), tumour neurosis Factor (TNFα), and granulocyte-macrophage colony-stimulating factor (GM-CFS). This study not only investigated the anti-inflammatory effects of oleocanthal in chondrocytes but also macrophages which is relevant as synovial macrophages generate the inflammatory cascade occurring in synovial fluid that leads to OA and RA [57]. Subsequently these authors suggest that there is a justification for oleocanthal to be developed as a therapeutic agent for future treatment of joint degenerative disease.

## 6. Oleocanthal and Brain Health

Ibuprofen has long been known to exert beneficial effects on markers of neuro-degenerative disease [58], therefore it was seen as logical to investigate oleocanthal’s role as a natural pharmacological agent, based on similarities between compounds in both perceptual and anti-inflammatory properties. Li and colleagues [23] have presented important findings demonstrating that oleocanthal inhibits tau fibrillization *in vitro* by forming an adduct with PHF6 peptide. PHF6, is a VQIXXK motif that resides in the microtubule binding region [59]. Common lesions that are observed in neuro-degenerative disease (*i.e.*, Alzheimer’s disease) are hyperphosphorylated tangles of tau and the PHF6 peptide enables the phosphorylation of tau. Therefore as oleocanthal modifies the PHF6 peptide it then disturbs tau-tau interaction and the subsequent fibril formation. Motivated to discover the mechanism by which oleocanthal reacts with the tau protein Monti and colleagues [24] reported that oleocanthal covalently modifies the construct of tau referred to as K18 in biologically relevant conditions [24]. Oleocanthal cross linked with two lysine residues and the end result was the rearrangement of the skeleton producing a more stable piridinium like complex [24].

Another type of lesion that are characteristic of Alzheimer’s disease are Β-amyloid peptides (Aβ) [60]. Derived from Aβ are diffusible ligands (ADDLs) which are neurotoxic factors believed to initiate the onset of Alzheimer’s disease [21]. *In vitro* evidence suggests that oleocanthal alters the structure of ADDLs and augments antibody clearance of ADDLs, therefore protecting hippocampal neurons from ADDL toxicity [21]. Of particular interest is the recent study conducted by Abuznait and colleagues [61]. This study importantly reported both *in vitro* and *in vivo* data indicating that oleocanthal enhances the clearance of Aβ by up regulating P-glycoprotein (P-gp) and also LDL lipoprotein receptor related protein-1 (LRPI). The authors focused on two peptide species that have been implicated in Alzheimer’s disease, both 1-Aβ_40_ and 1-Aβ_42_ mice brain endothelial cells. It was concluded that oleocanthal significantly enhanced the clearance rate of 1-Aβ_40_ in these cells. Further to that oleocanthal was tested for the first time *in vivo*. Intraperitoneal administration was conducted at 10 mg/kg/day of oleocanthal, twice daily over a duration of 2 weeks to wild type mice. Results showed that after administration of oleocanthal, the clearance rate of 1-Aβ_40_ was enhanced and moreover degradation of 1-Aβ_40_ was increased [61].

A recent cross sectional Australian study concluded that those suffering neurodegenerative disease showed a significantly lower adherence to a Mediterranean style dietary pattern [62], and there is a plethora of evidence research showing up to a 40% decrease in Alzheimer’s disease in populations consuming a Mediterranean style diet [63]. Perhaps oleocanthal, in conjunction with other phenolics, exerts a neuro-therapeutic potential that is reflected in the low incidence of neurodegenerative disease in populations that regularly consume the oil.

## 7. Further Considerations

The bioavailability of a nutrient is an important consideration when discussing potential health benefits. The bioavailability of the larger VOO phenolics, hydroxytyrosol, tyrosol and oleuropein has been confirmed (for review see [17]). Only one study has investigated the bioavailability of oleocanthal to date. Garcia-Villalba and colleagues [64] reported that largest concentration of metabolites produced and found in human urine were from oleocanthal, as well as hydroxytyrosol and oleuropein. This provides evidence of the metabolism of oleocanthal in the human body, however further studies are required to achieve an in depth understanding of the metabolism and bioavailability of this compound.

There are limitations when discussing the pharmacological actions of compounds. Certainly there is strong evidence that oleocanthal is an effective anti-inflammatory agent and demonstratespharmacological actions *in vitro*. However future *in vivo* studies are necessary to fully elucidate the potential of this compound as a pharmacological agent. There are many difficulties in extrapolating *in vitro* results to *in vivo* and caution must be taken when reporting the effects of a compound removed from the matrix in which it is normally contained. VOO phenolics function in a synergetic manner and complement each other in terms of anti-inflammatory, anti-oxidant, and anti-microbial properties, therefore oleocanthal, in addition to other VOO phenolics, plays a role in the health benefits associated with VOO intake.

## 8. Conclusions

In summary, a diet reflecting that which is consumed by the Mediterranean populace, incorporating daily VOO intake, has received significant attention over the last 40 plus years. Adherence to this dietary pattern is associated with a positive health profile and it would seem that VOO has beneficial effects on several health parameters. The phenolic fraction in particular has been highlighted to have beneficial anti-inflammatory effects in several studies. Since its discovery oleocanthal, a VOO phenolic, has gained a reputation of being a compound of interest in the goal of identifying therapeutic targets against many chronic inflammatory disease states including cancer, neurodegenerative, and joint degenerative disease. Oleocanthal efficiency in decreasing markers of arthritis, disrupting processes vital to the formation of Alzheimer’s and being a neuro-protective compound *in vitro* is confirmed. Furthermore oleocanthal reduces proliferation, migration and also promotes apoptosis in cancer cells as well as preventing tumour induced cell transformation. Oleocanthal’s bioavailability *in vivo* is not yet fully established, and this is important to access the pharmacological efficiency of oleocanthal. While there are many limitations associated with focusing on a compound outside the food matrix in which it is normally contained, it would seem that oleocanthal alone has significant pharmacological properties *in vitro* and is becoming recognised as a potential therapeutic agent.
